# Long-Term Effects of Adverse Maternal Care on Hypothalamic–Pituitary–Adrenal (HPA) Axis Function of Juvenile and Adolescent Macaques †

**DOI:** 10.3390/biology14020204

**Published:** 2025-02-15

**Authors:** Kai McCormack, Sara Bramlett, Elyse L. Morin, Erin R. Siebert, Dora Guzman, Brittany Howell, Mar M. Sanchez

**Affiliations:** 1 Department of Psychology, Spelman College, 350 Spelman Lane, Atlanta, GA 30314, USA; 2 Emory National Primate Research Center, Emory University, Atlanta, GA 30329, USA; sara.bramlett@emory.edu (S.B.);; 3 Department of Psychiatry & Behavioral Sciences, Emory University, Atlanta, GA 30322, USA; 4 Fralin Biomedical Research Institute at VTC, Virginia Tech, Roanoke, VA 24016, USA; 5 Department of Human Development and Family Science, Virginia Tech, Blacksburg, VA 24061, USA

**Keywords:** early life adversity, infant maltreatment, cortisol, dexamethasone, cross fostering, pubertal stress recalibration, nonhuman primate, rhesus monkey

## Abstract

Early life adversity (ELA) is a known risk factor for psychopathology, including stress-related anxiety and depressive disorders. The underlying mechanisms and developmental changes remain poorly understood. A likely underpinning is the impact of ELA on the development of stress response systems, including the hypothalamic–pituitary–adrenal (HPA) axis. Our group studied a translational ELA model of spontaneous infant maltreatment by the mother in rhesus macaques, where we used a cross-fostering design to randomly assign infant macaques to either Control or Maltreating (MALT) foster mothers at birth to examine the impact of adverse caregiving on the development of the HPA axis, while controlling for the confounding effects of heritable and prenatal factors. We previously reported higher levels of plasma and hair cortisol (CORT) across the first 6 postnatal months (equivalent to the first 2 years of life in humans) in the MALT than in the Control infants. Here, we followed the same cohort of infants longitudinally to assess the long-term developmental impact of this adverse experience on HPA axis function during the juvenile (12, 18 months) and late adolescent (~5 years) periods. For this, we collected measurements of diurnal CORT rhythm and glucocorticoid negative feedback using the dexamethasone suppression test (DST). At 12 months, we found higher diurnal CORT secretion in MALT females compared to Control females, and impaired negative feedback in response to the DST in both sexes in the MALT group. However, ELA group differences in the HPA axis function disappeared by 18 months and late adolescence, while sex differences in diurnal CORT rhythm emerged or became stronger. These results suggest that infant maltreatment causes dysregulation of the HPA axis during the first year of life, with HPA axis function normalizing later, during the pre-pubertal juvenile period and adolescence. This suggests that the impact of maltreatment on HPA axis function may be transient, at least if the adverse experience stops. Our findings are consistent with human evidence of recalibration/normalization of HPA axis function during adolescence in children that switch from adverse/deprived environments to supportive adoptive families. This research has broad implications regarding the biological processes that translate ELA to psychopathology during development and the pathways to resiliency.

## Simple Summary

Early life adversity, such as maltreatment and neglect, can increase the risk of developing psychiatric disorders later in life; this is likely due to the effects on the body’s developing stress systems. Here, we examined this potential biological mechanism in a nonhuman primate species, the rhesus monkey, where infant maltreatment by the mother leads to developmental consequences that are similar to those in humans. Our previous research found that infants with this early adverse experience had higher stress hormone (cortisol) levels during their first six months of life compared to non-maltreated animals. This study examined whether stress hormones were still elevated during the juvenile and adolescent years. At one year of age, maltreated females still showed elevated stress hormones, and all the maltreated animals demonstrated impaired regulation of cortisol secretion compared to the non-maltreated animals. However, the effects of the early adverse experience on stress hormone secretion disappeared in adolescence, while in adulthood typical sex differences in stress hormone secretion emerged. These findings suggest that the effects of early maltreatment on stress hormones may be transient, at least if the adverse experience stops. Understanding how our biology responds to, adapts to, and recovers from early life stress could help us develop better treatments for people affected by childhood adversity.

## 1. Introduction

Childhood maltreatment (MALT), a form of early life adversity (ELA), is associated with the development of long-term negative health outcomes [1,2]. It is a major risk factor for the development of psychopathologies, including depressive and anxiety disorders, inflammation, substance use disorders, and cognitive and behavioral deficits [3]. Despite this established connection, the neurobiological mechanisms of this relationship and the developmental trajectories of change in the critical neuroendocrine systems affected remain poorly understood.

ELA can impact the development of physiological stress systems, including the hypothalamic–pituitary–adrenal (HPA) axis. The HPA axis is a key homeostatic neuroendocrine system with a basal circadian rhythm of glucocorticoid (GC) secretion as well as a main role in the neuroendocrine stress response [4]. Basal GC secretion (cortisol (CORT) in primates; corticosterone in rodents) is tuned to meet the perceived metabolic demands of the day, whereas stress-induced GC elevations function to activate catabolic processes to mobilize and provide energy substrates to organs in high energetic need during the stress response, thereby promoting survival. At elevated concentrations, GCs exert physiological effects predominantly through binding to the glucocorticoid receptor (GR), which acts as a transcription factor in a variety of tissues to control the expression of genes involved in immune function, metabolism, arousal, fluid homeostasis, and, importantly, neural development and cognition [3]. Hypothalamic, pituitary, and limbic GRs are also critical to shut down stress-induced HPA axis activation through GC-mediated negative feedback mechanisms [5,6,7].

Research across several species (e.g., rodents, nonhuman primates (NHPs), and humans) suggests that chronic stress can affect both the baseline, stress-induced response and the negative feedback functions of the HPA axis [5]. However, the directionality of effects reported is inconsistent, with chronic stress having been linked with increased levels of basal GC release [8,9], as well as with blunted basal GC levels [4,10]. Habituation of the HPA axis, or hyporesponsiveness, has been commonly reported in response to repeated exposure to the same chronic stress [5,11,12]. When exposed to novel stressors, however, the HPA axis stress response can be maintained or can even hyper-respond [12,13]. In terms of HPA axis negative feedback, repeated exposure to the same stressor has been linked to habituation of the HPA axis, resulting in decreased secretion of GCs over time [5,14], while other studies report an increase in baseline GC secretion, which is likely due to the loss of GC negative feedback control [8,9]. It is important to note that the effects on the HPA axis basal, stress response, and negative feedback functions vary depending on the intensity and frequency of the chronic stressor(s), their timing in life, and genetic and environmental factors [5,15].

The literature is also mixed on *how* ELA affects the development of the HPA axis. An interesting hypothesis suggests that repeated stress/adversity early in life might cause prolonged HPA axis overactivation and hypercortisolemia, which over time induces compensatory downregulation of the axis, leading to basal hypocortisolemia and flattening of the diurnal rhythm [4,15,16], in parallel to, and potentially directly contributing to, blunted emotional and sympathetic reactivity to the threats reported as some of the sequelae of MALT [17,18]. ELA has been reported to alter the maturational trajectory of the HPA axis [19], as well as connectivity between brain regions involved in stress and emotional regulation, such as the amygdala and prefrontal cortex (PFC) [20,21,22,23]. Chronic exposure to high levels of GCs due to repeated activation of the HPA axis early in life could underlie these neurodevelopmental changes [24,25,26,27,28]. In addition, hyperactive HPA axis function can have deleterious physiological consequences in other systems, such as unchecked inflammation, metabolism, growth and sleep disturbances, which precipitate psychopathologies [29,30]. Despite all of these hypotheses, and conflicting findings, the effects on basal HPA axis activity and rhythmicity, as well as on its negative feedback, remain relatively understudied, particularly in studies using prospective, longitudinal designs during development. This is germane because aberrant basal diurnal CORT rhythm is associated with a variety disease states, including psychiatric disorders such as depression and post-traumatic stress disorder (PTSD) [31,32]. A complication in human ELA studies is that they are often retrospective and focus on subjects in which psychopathology is already manifest, typically adults and sometimes adolescents. Prospective, longitudinal studies in humans are very challenging, and it is often difficult to disentangle the long-term effects of postnatal adverse experiences/stress at explicit developmental stages from the effects of heritable and prenatal experiences and the effects of confounding factors beyond the ELA (i.e., diet, socioeconomic status, and exposure to drugs).

Socially housed rhesus macaques are a frequently used translational model for studying ELA and developmental outcomes; they allow more experimental control of confounding variables, while also being quite similar to humans in terms of their biological, physiological, neural and genetic relatedness, complex social behaviors and interactions, and strong mother–infant bonds. Of special note is the fact that naturally occurring maternal MALT has also been reported in several species of nonhuman primates (NHPs), including rhesus monkeys, in both captive and non-captive settings [33,34]. Maternal MALT in rhesus macaques is defined as early life physical abuse and rejection of the infant by the mother, typically during the first three months of life, resulting in infant distress and elevations in stress hormones [34,35]. Infant maltreatment, including physical abuse and neglect, is uncommon in NHP mothers, with rhesus macaque rates estimated to be 2–5% among mothers [34,36,37,38].

Our findings are consistent with human evidence linking MALT with long-term impairments in stress and emotion regulation in children [39,40], and our group has reported that infant MALT in rhesus monkeys also leads to increased emotional reactivity, anxiety-like behaviors, hyperactivity of stress neuroendocrine systems, and peripheral inflammation during the infant and juvenile periods [37,41,42,43,44]. Some of these early infant alterations have been associated with alterations in brain regions that control emotional and stress responses, including increased amygdala volumes and alterations in cortico-limbic connectivity [42,45,46]. However, a critical question in the field is the following: how much are these alterations due to postnatal early adverse caregiving and how much are they due to heritable genetic/epigenetic factors or prenatal programming? That is, how much is due to nature versus nurture?

Our lab has recently attempted to disentangle the effects of ELA due to maternal MALT from those of heritability on the development of social and HPA axis function in rhesus macaques. To carry this out, we used a cross-fostering design, in which infants were randomly assigned to be fostered by either competent (Control) mothers or mothers with a history of infant MALT at birth [35,45]. Across the first 6 postnatal months, the fostered MALT infants exhibited greater emotional reactivity and higher levels of basal plasma and hair CORT levels compared to the Controls [44]. These findings replicate previous reports by our group of elevated CORT levels in biological MALT (non-cross-fostered) offspring [47] and in other rhesus macaque models of ELA, including higher levels of hair CORT at month 6 in rhesus infants who experienced peer-rearing, compared to mother-reared infants [48]. These results suggest that ELA caused by infant MALT postnatal experience increases the rhesus infant’s HPA axis activity, which is detectable across the first six months as both plasma (acute measurement) and hair (chronic measurement) CORT and may have long-term consequences for physiological development.

The goal of the present study [49] was to examine the long-term maturational impact of infant MALT on the HPA axis function of cross-fostered animals, beyond the first six months of life, after MALT stops and into the juvenile and post-pubertal, late adolescent years. We were also interested in examining sex differences. In humans, there is gender bias in psychopathology prevalence, with women being at a greater risk for stress-related disorders such as depression and anxiety disorders [50], which are particularly likely to emerge during adolescence [51]. While psychosocial factors may also be at play, extensive cross-talk between the hypothalamic–pituitary–gonadal (HPG) and HPA axes likely constitute a physiological component of this disparity. Surprisingly, there is a scarcity of literature on sex-specific differences in circadian HPA axis rhythmicity in the transition from the juvenile pre-pubertal to post-pubertal adolescent periods [52,53], let alone on the interaction with early life experience [53,54].

In sum, mounting evidence suggests that periods of chronic stress early in life might cause prolonged HPA axis overactivation and hypercortisolemia, which over time induce compensatory downregulation of the axis, leading to basal hypocortisolemia and flattening of the diurnal rhythm [4,15,16]. We test this hypothesis here, examining the diurnal rhythm of basal plasma CORT longitudinally to determine the timing of HPA axis reprogramming throughout rhesus development. The molecular mechanisms of ELA-induced HPA axis dysregulation are still unclear, but several studies point to changes in GR-mediated negative feedback to the axis [55,56,57]. In humans, both basal hypercortisolemia and impaired negative feedback are found in patients with major depressive disorder, while basal hypocortisolemia and enhanced negative feedback are reported in patients with PTSD [58]. Thus, as a corollary aim of our study, we also investigate the sensitivity of GR-mediated negative feedback to the axis as it relates to basal HPA axis activity. Negative feedback is determined using the dexamethasone suppression test (DST), a pharmacological challenge using synthetic GC administration. Following the primary hypothesis, we expected the predicted switch from HPA axis hyperactivity to hypoactivity to be accompanied by GR downregulation, evidenced by impaired CORT suppression during the DST, followed by super-suppression.

## 2. Materials and Methods

### 2.1. Subjects and Housing

A total of 44 rhesus macaques (*M. mulatta*) and their mothers were studied from the early juvenile stage (12 months of age) through to late adolescence (~4.5–5.5 years), as part of a larger project examining the developmental consequences of infant MALT from birth to adulthood [35,44,45,46]. Of the 44 subjects, 22 experienced competent maternal care (Control: 11 males, 11 females) and 22 experienced maternal MALT (14 males, 8 females). The subjects lived in large social groups at the Emory National Primate Research Center (ENPRC) Field Station, Emory University (Lawrenceville, GA, USA), until adolescence (approximately 4 years), when 25 subjects selected to continue testing in a related study were relocated to the ENPRC Main Center (Atlanta, GA, USA). The animals transferred to the Main Center were pair-housed with familiar, same-sex partners from the same social group to maintain established social ranks and were counterbalanced with the experimental group. At the ENPRC Main Center the animal facility maintains an ambient temperature of 22 ± 2 °C with 25–50% humidity, and lighting is based on a 12 h light/dark cycle (lights on: 0700; lights off: 1900). Environmental enrichment, such as toys, were provided in the home cage on a regular basis. After several months of acclimation to the new housing and environment, the animals underwent neuroendocrine assessments of HPA axis function. At both locations, the subjects were provided access to food twice daily, including standard high-fiber, low-fat monkey chow (Purina Mills LLC, St Louis, MO, USA), fruits, and vegetables, and water ad libitum. All procedures were performed in accordance with the Animal Welfare Act and the U.S. Department of Health and Human Services “Guide for the Care and Use of Laboratory Animals” and approved by the Emory Institutional Animal Care and Use Committee (IACUC).

### 2.2. Cross-Fostering Procedures and Group Classifications

The subjects were born of multiparous mothers with either a consistent history of competent maternal care (Control) or of infant maltreatment (MALT). Selection of mothers for assignment to the study was based on existing records in our lab of their maltreating or competent care of prior infants, which are very stable maternal traits [37]. Indeed, maltreating rhesus mothers tend to maltreat all their infants with similar patterns and rates [37], and these traits are atypical and transmitted across generations along the maternal line [38]. This historical information regarding maternal care patterns has been collected by our group over the past 20 years for our ongoing work. In addition, the animal care staff at the ENPRC closely monitor newborn infants in their group across the first month of life to ensure that they are thriving, and they are also able to identify potential study subjects using their data. Altogether, there are abundant animal records on competent versus multigenerational maltreatment, as well as behavioral data on the mothers’ interactions with prior infants.

In order to disentangle the effects of early adverse caregiving experiences from those of heritable and prenatal factors, a cross-fostering design was used, with a random assignment of infants to either Control or MALT foster mothers following published procedures [35,45]. The infants were cross-fostered on the first day of life (except for 2 infants cross-fostered within 48–72 h of birth). Only MALT mothers with a history of infant maltreatment that was not severe were selected due to animal welfare ethical standards. Control mothers were selected from those with a history of competent maternal care and opportunistically for giving birth in temporal proximity to a MALT mother.

Of the 22 infants raised by Control mothers, 12 were born to biological Control mothers (7 males, 5 females) and 9 to biological MALT mothers (3 males, 6 females), and 1 male was raised by its biological mother (i.e., non-cross-fostered; it was added to supplement the study during adolescence and thus was not included in any of the infant or juvenile studies). Of the 22 infants raised by MALT foster mothers, 12 were born to biological Control mothers (9 males, 3 females) and 10 to other biological MALT mothers (5 males, 5 females). Groups were counterbalanced for social dominance rank (high, middle, low).

To verify maternal MALT, or the lack thereof, mother–infant pairs were surveyed in their social groups during 30 min infant focal observations, 5 days/week, for the first 4 postnatal weeks, followed by observations 2 days/week in month 2, and 1 day/week during months 3–6, which totaled 44 × 30 min observations (22 h) per mother–infant pair. This data collection schedule is optimal to document infant MALT in rhesus monkeys, operationalized in this study as co-morbid physical abuse and rejection of the infant by the mother in the first 3 months of life, following the criteria described in previous publications [35,41,44,45,59]. Briefly, physical abuse was operationally defined as violent infant-directed behaviors by the mother, which are aberrant, cause infant distress, and include dragging, crushing, throwing, stepping or sitting on, and rough grooming. These behaviors are co-morbid with early bouts of infant rejection, operationalized as the mother preventing contact or infant access to her ventrum by pushing the infant away or blocking it with her arm or twisting her torso away and also causing infant distress. Infant MALT (physical abuse co-morbid with rejection by the mother) causes emotional distress and elevations in stress hormones in infant rhesus monkeys through 6 months of age [4,34,35,41,42,44]. Control mothers exhibit species-typical competent maternal care (e.g., nursing, cradling, protection, ventral contact, and grooming of infant) and do not exhibit physical abuse. The infants were monitored closely for trauma resulting from any observed physical abuse; however, veterinary care was not needed for any infant. Behavioral data were collected between 0700 and 1100 and with an inter-observer reliability threshold of >90% agreement and a Cohen’s kappa exceeding 0.8.

### 2.3. Training and Capture Procedures

Juvenile macaques (12 and 18 months) are relatively independent of their mothers; thus, using positive reinforcement, the subjects were trained to separate from the social group in the outdoor enclosure and move into an indoor room and then into a specialized transfer box with a squeeze mechanism. From the transfer box, the subjects were trained to present a leg for an awake blood sample collection through saphenous venipuncture. At adolescence, when the subjects were housed at the ENPRC Main Center, they were trained again to transfer from their home cage to the specialized transfer box and to present a leg for an awake blood sample collection through saphenous venipuncture. Blood samples were collected within 10 min from disturbance of the compound or animal room to reflect baseline plasma CORT levels, as was previously demonstrated to be effective [16,41,43,44,60].

### 2.4. HPA Axis Basal Activity: Diurnal CORT Secretory Rhythm

HPA axis basal activity was measured at 12 months, 18 months (pre-puberty), and adolescence (post-puberty) (4.5–5.5 years). Because the subjects lived under natural light conditions that determined their circadian CORT secretory rhythms while housed at the ENPRC Field Station, time points were selected based on daylight times published by the US Naval Observatory (http://aa.usno.navy.mil/data/docs/RS_OneYear.php, accessed on 25 January 2023) for juvenile HPA axis assessments at 12 and 18 months. At those ages, the basal diurnal CORT secretory rhythm was examined by drawing basal blood samples collected at sunrise (AM), sunset (NITE), and midway between sunrise and sunset (PM), as previously published [43,61]. During adolescence, the subjects lived under artificial lighting conditions set to the clock time at the ENPRC Main Center; thus, the basal diurnal CORT secretory rhythm was examined via basal blood samples collected at 0730 h (AM), 1245 h (PM), and 1800 h (NITE) (30 min after lights on, half-way lights on/off, and 1 h before lights off, respectively), following the published protocols by our group that match diurnal CORT secretion under natural light conditions [61]. Female adolescent samples were collected during the follicular phase (5–10 days after detection of menses), when gonadal steroids are typically at their lowest levels. All blood samples were collected in pre-chilled vacutainer tubes containing EDTA, immediately placed on ice, and centrifuged for 15 min at 300 RPM and 4 °C. Plasma was aliquoted into sterile microcentrifuge tubes and stored at −80 °C until the assay.

### 2.5. Plasma Cortisol Assay

Plasma CORT assays were performed by the ENPRC Biomarkers Core Laboratory. Plasma was thawed at 4 °C, and 20 µL of plasma was analyzed by UPLC-ESI-tandem mass spectrometry using an AB Sciex TripleQuad 6500 (AB Sciex LLC, Framinham, MA, USA) following previously published protocols [44]. CORT concentrations for each sample were calculated using linear regression analysis of a standard curve. The quantification range for the assay was 0.01–100 µg/dL cortisol, with calibration standards prepared at concentrations of 0, 0.1, 0.5, 1, 5, 10, 20, 50, 75, 100 µg/dL for each run. Three fortified quality control (QC) samples were also analyzed in duplicate in each run. The intra- and inter-assay precision values [percentage coefficient of variation (CV%)] were <5% and <7%, respectively.

### 2.6. Glucocorticoid-Mediated Negative Feedback

A dexamethasone suppression test (DST) was administered to the subjects at 12 and 18 months of age, as well as during adolescence. Dexamethasone (DEX) is a synthetic GC with a high affinity for GR; it is administered at night in the DST to suppress the CORT levels the morning after to examine GC negative feedback sensitivity of the HPA axis [58,60,62,63,64]. The subjects were injected with DEX (0.25 mg/kg i.m.) 11 h prior to the morning blood sample, as previously used in macaques to keep CORT levels suppressed below 5 ug/dL and still detect differences in GC sensitivity between animals with different stress exposure or developmental manipulations [60,62,65]. The following day, blood samples were collected in the morning (AM) and at midday (PM) following the protocols described above and the DEX suppression tests previously used by our group [65]. These two time points were used to evaluate CORT DEX suppression (AM) and escape (PM) by comparing them with the sunrise and midday CORT levels from the diurnal CORT rhythm assessments at the same age.

### 2.7. Data Analysis

Of the blood samples collected for diurnal CORT rhythm at all ages, 11 were excluded from analysis (8 for collection >10 min from disturbance time; 3 were duplicates of samples eliminated at random). Of the blood samples collected for DST at all ages, 9 were excluded from analysis (8 for collection >10 min from disturbance time, 1 due to subject illness during the access). SPSS, v29, was used for all statistical analyses.

Linear regressions were first used to examine whether time from disturbance affected plasma CORT in order to control for this confounding factor in the statistical models at each age if needed. Because no significant correlations were detected at any time point, time from disturbance was not included as a covariate in the subsequent analyses.

Analyses of covariance (ANCOVAs) were used to examine the effects of caregiving experience (Control, MALT), including the biological mother group (Control or MALT) as a covariate to control for any residual genetic effects. All analyses were first run with sex (male, female) as an additional between-subjects factor, which was eliminated from the final analysis if no effect of sex was found. Sex was therefore included in all 3 diurnal CORT analyses, but not in the DST ANCOVA statistical models. The significance level was set at *p* < 0.05 for all analyses.

Diurnal CORT rhythms at each age were examined using repeated measures ANCOVAs with time of day (3 levels: AM, PM, NITE) as the within-subjects factor and maternal care (2 levels: Control, MALT) and sex (2 levels: male, female) as the between-subjects factors. DST results at all ages were examined using repeated measures ANCOVAs with two within-subjects factors, DEX (2 levels: −DEX, +DEX) and time of day (2 levels: AM, PM), and one between-subjects factor, maternal care (2 levels: Control, MALT). Significant interactions were further evaluated using Bonferroni-adjusted post hoc comparisons of the estimated marginal means in SPSS. Means (±SEM), adjusted for the biological mother group, are included for significant post hoc interactions.

## 3. Results

### 3.1. HPA Axis Basal Activity

Upon examination of the diurnal CORT rhythms at all three ages, we found the expected significant main effect of time of day on plasma CORT (12 months: *F*_(2,50)_ = 3.30, *p* < 0.05, η^2^*_p_* = 0.12; 18 months: *F*_(2,56)_ = 14.19, *p* < 0.001, η^2^*_p_* = 0.34; adolescence: *F*_(2,38)_ = 22.72, *p* < 0.001, η^2^*_p_* = 0.55), with values decreasing from morning (AM) to evening (NITE).

**Figure 1:**
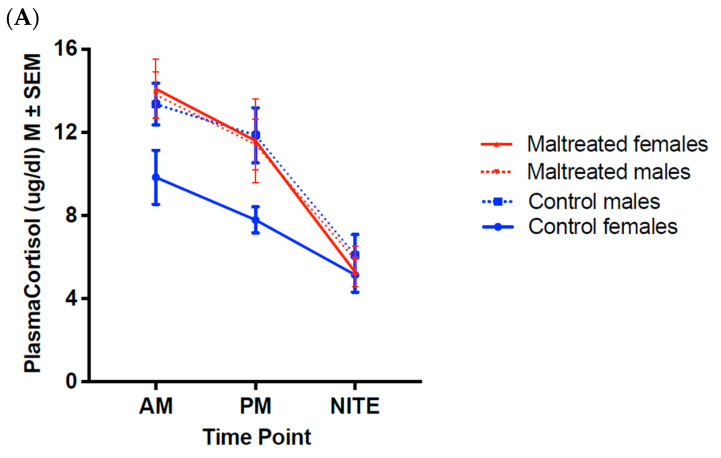

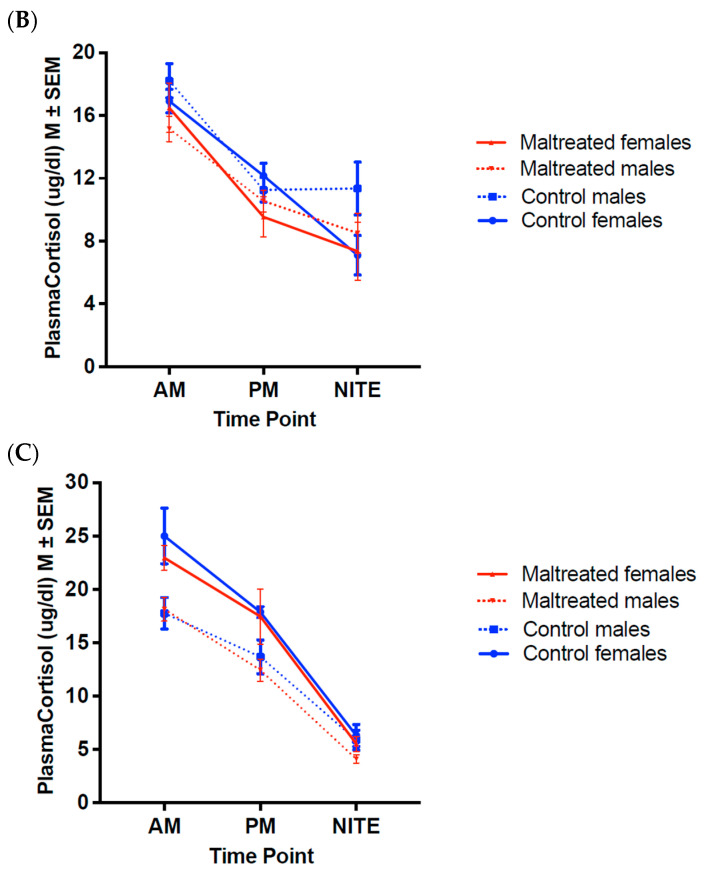
Diurnal CORT rhythms during the juvenile and adolescence periods. (Mean ± SEM). (**A**) Twelve months: Significant time effect (*F*_(2,50)_ = 3.03, *p* < 0.05), with CORT levels decreasing across the day for all groups and maternal care x sex effect (*F*_(1,25)_ = 4.78, *p* = 0.04), with post hoc tests revealing maternal care effects on females (*F*_(1,18)_ = 6.37, *p* = 0.02) but not males (*F*_(1,18)_ = 0.08, *p* = 0.78). (**B**) Eighteen months: significant time effect (*F*_(2,56)_ = 14.19, *p* < 0.001), with CORT levels decreasing across the day and time of day × sex interaction effect (*F*_(2,56)_ = 3.72, *p* = 0.03). (**C**) Adolescence: Significant time effect (*F*_(2,38)_ = 22.72, *p* < 0.001), with CORT levels decreasing across the day, sex effect (*F*_(1,19)_ = 15.54, *p* < 0.001), and time of day × sex interaction effect (*F*_(2,38)_ = 5.48, *p* = 0.008), with post hoc tests revealing higher CORT in females than males at the AM (*F*_(1,19)_ = 12.90, *p* = 0.002) and PM time points (*F*_(1,19)_ = 9.65, *p* = 0.006, η^2^*_p_* = 0.34).

At 12 months, there was a significant maternal care × sex interaction effect (*F*_(1,25)_ = 4.78, *p* = 0.04, η^2^*_p_* = 0.16) (Figure 1A). The post hoc analyses revealed that maternal care influenced diurnal CORT rhythm in females (*F*_(1,18)_ = 6.37, *p* = 0.02, η^2^*_p_* = 0.20) but not in males (*F*_(1,18)_ = 0.08, *p* = 0.78, η^2^*_p_* = 0.003). MALT females had higher CORT values across the day compared to Control females, while there was no difference across the day between MALT males and Control males.

At 18 months, there was a significant time of day × sex interaction effect (*F*_(2,56)_ = 3.72, *p* = 0.03, η^2^*_p_* = 0.12) (Figure 1B). In the post hoc analyses, the effect of sex did not reach significance at any time point. No other significant effects were found at this age.

During adolescence, there was a significant main effect of sex (*F*_(1,19)_ = 15.54, *p* < 0.001, η^2^*_p_* = 0.45) and a time of day × sex interaction effect (*F*_(2,38)_ = 5.48, *p* = 0.008, η^2^*_p_* = 0.22) (Figure 1C). The post hoc analyses revealed a significant difference in CORT values at the AM time point (*F*_(1,19)_ = 12.90, *p* = 0.002, η^2^*_p_* = 0.40), with females having higher plasma CORT (AM: *M* = 24.49.9 ± 1.32 μg/dL) than males (AM: *M* = 17.68 ± 1.26 μg/dL). The same effect was found for the PM time point (*F*_(1,19)_ = 9.65, *p* = 0.006, η^2^*_p_* = 0.34), with females again having higher plasma CORT (PM: *M* = 17.85 ± 1.13 μg/dL) compared to males (PM: *M* = 12.83 ± 1.08 μg/dL). These differences vanished by NITE (*F*_(1,19)_ = 0.78, *p* = 0.39, η^2^*_p_* = 0.04).

### 3.2. Glucocorticoid-Mediated Negative Feedback

**Figure 2:**
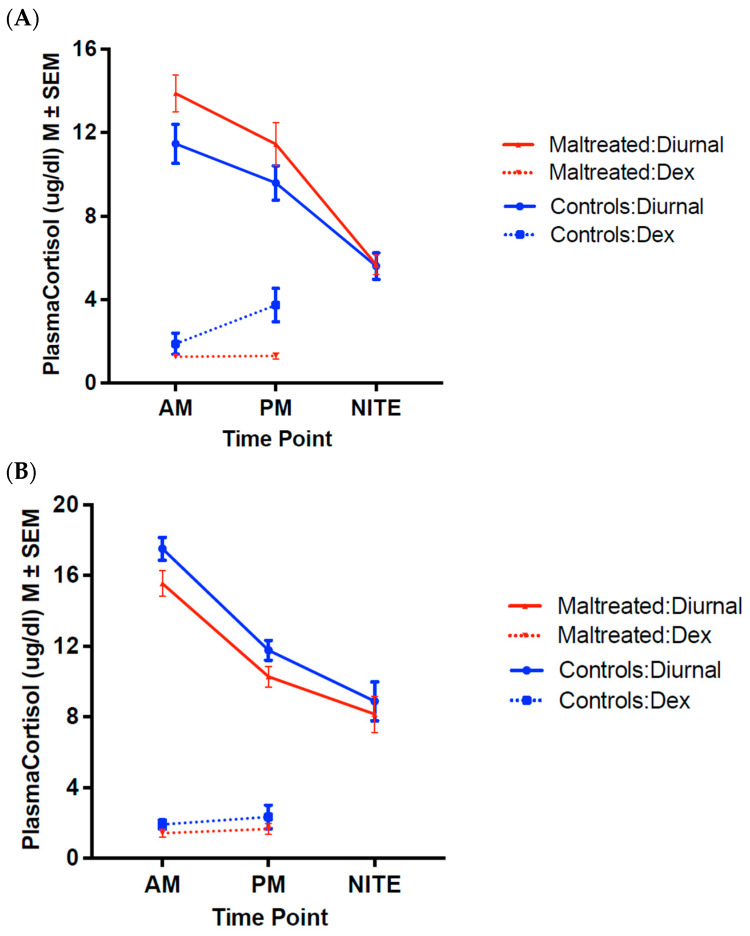
Dexamethasone suppression of diurnal CORT (−DEX) secretion at 12 and 18 months (juvenile period). (Mean ± SEM). (**A**) Twelve months: Significant DEX effect with lower CORT values in all groups under +DEX compared to −DEX *F*_(1,23)_ = 17.78, *p* < 0.001), time of day × DEX interaction effect (*F*_(1,23)_ = 10.37, *p* = 0.004), with levels decreasing from the AM to PM time points under the −DEX condition; however, in the +DEX condition values increased from the AM (suppressed CORT) to PM (escape) time points. A maternal care × DEX interaction effect was also found (*F*_(1,23)_ = 8.50, *p* = 0.008) with post hoc analyses revealing that this effect was driven by lower CORT levels across the day in MALT than in Control animals in the +DEX condition (F_(1,23)_ = 4.96, *p* = 0.04). (**B**) Eighteen months: Significant DEX effect with lower CORT values in all groups under +DEX compared to −DEX (*F*_(1,30)_ = 186.77, *p* < 0.001) and a time of day × DEX interaction effect (*F*_(1,30)_ = 8.74, *p* = 0.01), with values decreasing from the AM to PM time points under the −DEX condition, but remaining the same (suppressed) at AM and PM time points in the +DEX condition. In contrast to 12 months, no maternal care × DEX interaction effects were detected at this age. Solid lines represent basal diurnal CORT levels (−DEX condition), dashed lines represent CORT levels after DEX treatment (+DEX condition).

As would be expected, there was a main effect for DEX, with the CORT values being significantly lower in all groups under +DEX compared to −DEX conditions (12 months: *F*_(1,23)_ = 17.78, *p* < 0.001, η^2^*_p_* = 0.44; 18 months: *F*_(1,30)_ = 186.77, *p* < 0.001, η^2^*_p_* = 0.86). Additionally, there was a time of day × DEX interaction at both ages (12 months: *F*_(1,23)_ = 10.37, *p* = 0.004, η^2^*_p_* = 0.31; 18 months (*F*_(1,30)_ = 8.74, *p* = 0.01, η^2^*_p_* = 0.23)), with CORT levels decreasing from the AM to PM time points under the −DEX condition for both ages; however, in the +DEX condition, the CORT values at 12 months increased from the AM (suppressed CORT) to PM (escape) time points, while remaining the same at 18 months (Figure 2A,B).

At 12 months, we also found a significant maternal care × DEX interaction effect (*F*_(1,23)_ = 8.50, *p* = 0.008, η^2^*_p_* = 0.27) (Figure 2A). The post hoc analyses revealed that this effect was being driven by the fact that across the day, the CORT levels were significantly lower in the MALT (*M* = 1.26 ± 0.45 μg/dL) than the Control animals (*M* = 2.73 ± 0.48 μg/dL) in the +DEX condition (*F*_(1,23)_ = 4.96, *p* = 0.04, η^2^*_p_* = 0.18).

At 18 months, there were no maternal care × DEX interaction effects. Thus, the differential effect of maternal care on recovery from DEX suppression found at 12 months was no longer present (Figure 2B). Although there was a main effect of maternal care (*F*_(1,30)_ = 5.01, *p* = 0.03, η^2^*_p_* = 0.14) on CORT values, these results should be viewed with extreme caution as they are based on the average CORT collapsed across the two DEX conditions. (−DEX, +DEX) and across both AM and PM time points, and they are not relevant to understanding HPA axis reactivity across time.

DEX treatment at adolescence resulted in a ceiling effect of CORT suppression with no escape at midday in any animal from any experimental group or sex. Thus, although significant effects of DEX (*F*_(1,18)_ = 200.342, *p* < 0.0001, η^2^*_p_* = 0.918) and DEX × time were detected (*F*_(1,18)_ = 15.6, *p* < 0.002, η^2^*_p_* = 0.464), these statistical results and the lack of a main effect of maternal care or time of day or interaction effects should be interpreted with caution, and the results are not included here or in Figure 2.

## 4. Discussion

One of the central questions that this study aimed to address was how ELA, specifically in the form of infant maltreatment, impacts basal HPA axis function and negative feedback. A previous publication on this cohort of animals [44] demonstrated initial basal hyperactivity (hypercortisolemia) across the first six months of life in MALT infants, aligning with the period of sustained maltreatment (adverse experience). Based on previous studies [4,15,16], we hypothesized an eventual reversal to hypocortisolemia during the juvenile and adolescent years in the absence of adversity. Contrary to our predictions, we detected persistent basal hypercortisolemia at 12 months in MALT females compared to Control females, with elevated CORT secretion across the diurnal rhythm and a steeper diurnal slope than Control females. The CORT diurnal secretory rhythm was similar in both Control and MALT males. Maltreatment-related effects on basal CORT levels disappeared by 18 months and the HPA axis function normalized at the adolescent time point. Thus, the disruption induced by infant maltreatment of basal HPA axis function persists past the initial period of infant ELA exposure through the first year of life, as evidenced by alterations in diurnal CORT rhythm in MALT females and impaired glucocorticoid-mediated negative feedback in the MALT group. However, MALT differences in the HPA axis function seem transient, as they disappeared by 18 months and late adolescence, while sex differences emerged or became stronger. These results suggest that HPA axis function normalizes during the pre-pubertal juvenile period and adolescence, at least in the case of maternal MALT and if the adverse experience stops. Our findings are consistent with human evidence of recalibration/normalization of the HPA axis function during adolescence in children that switch from adverse/deprived environments to supportive adoptive families [66]. This research has broad implications regarding the biological processes that translate ELA to psychopathology during development and pathways to resiliency. It suggests that the impacts on the HPA stress response are not permanent and may even be attenuated with cessation of the disruption to normative maternal care, providing a strong rationale for supporting parenting interventions, particularly in those at risk for maternal maltreatment.

This infant maltreatment ELA experience leads to long-term HPA axis hyperactivity and dysregulation (impaired/super-responsive GC-mediated negative feedback) during the first year of life (infancy: 35, 44; and early juvenile period (12 months): this manuscript), as well as behavioral signs of distress, emotional hyperreactivity, and anxiety through adolescence [44,59]. However, despite the persistent impairments in emotional regulation, HPA axis function appears to normalize during the pre-pubertal juvenile period (18 months) and adolescence, suggesting that the impact of maltreatment on HPA axis function may be transient, at least in the case of maternal MALT. Unlike human studies, infant rhesus monkeys experience MALT during a narrow time period during infancy, which predominately occurs during the first three-six months of life [44]; this is equivalent to the first 1–2 years of life in humans, with other alterations in maternal care and the mother–infant relationship extending across the first year.

In contrast, childhood maltreatment can be a much more prolonged experience in humans, lasting through adolescence and followed by lifetime revictimization, which is a common phenomenon reported in individuals that experience childhood maltreatment in human studies [67,68], potentially leading to different long-term impacts on HPA axis function. In addition, human victims of childhood maltreatment undoubtedly experience additional social stressors that cannot be replicated in this animal model. However, our findings regarding HPA axis function normalization during the pre-pubertal and adolescence rhesus stages are consistent with human evidence of recalibration/normalization of HPA axis function during adolescence in children that switch from adverse/deprived environments to supportive adoptive families [66]. Taken altogether, these findings in both humans and NHP species have broad implications regarding the developmental course and biological processes that translate ELA to psychopathology during development and potential pathways to resiliency. Regardless of the transient nature of maltreatment-induced alterations in basal HPA axis activity, elevated GC exposure during infancy and the early juvenile period can have lasting effects by biasing downstream neurodevelopment. For instance, exogenous GC treatment and GR level manipulations in the brain early in life have been shown to alter limbic function and to lead to a predisposition for depressive- and anxiety-like behaviors [69,70].

Unlike other primate models of ELA, such as peer- and surrogate-rearing, our MALT animals remain in large social groups, and continuously have interactions with peers, family members, and other adults. In other words, we may be detecting a normalization/recalibration of HPA axis function in our animals with ELA experience at later ages due to other social experiences and factors that buffer the long-term consequences of adverse caregiving. The few NHP studies that have examined the long-term effects of ELA on HPA axis activity beyond the first year of life, mainly intermittent maternal separation and peer- and surrogate-rearing, are conflicting; this is likely based on the types of experiences, as well as the timing and the measurements taken. For example, intermittent mother–infant separations had profound effects on the mother–infant bond, the tempo of social development and anxiety and was associated with flat diurnal CORT rhythms [16]. Feng and colleagues [71] reported that peer-reared rhesus macaques demonstrated lower levels of hair CORT when they were 2 and 3.5 years old compared to mother-reared animals, despite no differences in basal plasma CORT levels at year 2. Another study which examined hair CORT across the first two years of life in mother-, peer-, and surrogate-reared rhesus macaques, reported variability across the first 18 months, with surrogate-reared animals having the highest CORT levels at months 12 and 18, followed by the peer-reared and then the mother-reared animals; however, by 2 years of age, all groups had similar hair CORT concentrations [48]. Another study that compared basal plasma CORT levels of mother-, peer-, and surrogate-reared animals did not find group differences at 14, 26, 37, or 49 months of age, either [72]. The inconsistencies in these findings suggest that there are other factors not being accounted for that will need to be defined and measured to obtain a comprehensive mechanistic understanding of how different forms of ELA impact development.

An important point for this discussion is that even if the HPA axis function of maltreated macaques normalizes by 18 months of age, long-term impacts in other systems have already occurred. That is, exposure to high basal diurnal CORT levels (and possibly also during the night, although we do not know because we could not collect plasma throughout the full circadian rhythm) for the first 12 months of life [44], could lead to alterations in physiological and metabolic function and in brain structural and functional development. Indeed, we have previously published associations between high infant levels of CORT and alterations in amygdala connectivity [46]. Neural structural and functional impacts of CORT can be explained by its impact on developmental processes, such as myelination, neuronal arborization, synaptogenesis, and pruning through glucocorticoid receptor (GR)- and mineralocorticoid receptor (MR)-mediated effects on gene expression [24,25,26,27]. Alterations in neurodevelopment could lead to long-term, persistent alterations in the behavior and cognition of maltreated animals, including impaired emotional regulation previously reported in this cohort, which showed higher emotional reactivity and anxiety from infancy through to adolescence [44,59].

This study also investigated alterations in GC-mediated negative feedback as a potential mechanism of maltreatment-induced HPA axis dysregulation. Chronic HPA axis overactivation can lead to eventual downregulation of the axis, including lower GR expression and available protein, in an effort to maintain physiological homeostasis [4,15,16]. At 12 months, MALT subjects exhibited super-suppression of next-day CORT secretion following DEX treatment. This was an unexpected finding for two reasons. First, CORT super-suppression following the DST reflects—and is interpreted as—hypersensitivity of GRs to GC-mediated negative feedback or higher GR expression levels, which is typically thought to occur in response to basal hypocortisolemia and has been reported in humans with PTSD [63,64,73], but not to the sustained hypercortisolemia found in MALT animals through 12 months. Although hypercortisolemia has also been found in parallel with DST CORT super-suppression in macaques with neonatal amygdala lesions [60], the findings are difficult to explain, unless it is due to a compensatory mechanism. Second, the CORT super-suppression following the DST was found in both sexes, while the elevated diurnal CORT secretion was only detected in MALT females. In any case, further studies will be required to elucidate the underlying mechanism behind the GR hypersensitivity found in parallel with basal diurnal CORT hypersecretion in MALT macaques. By 18 months, both the Control and MALT subjects exhibited the same magnitude of CORT suppression during the DST, indicating another change in HPA axis function that persists past the initial period of infant MALT and yet may be transient.

On the topic of sex differences, this study revealed an elevated diurnal CORT secretory rhythm in only the MALT females, not the males, in comparison to the Control females at 12 months, as well as in all adolescent females compared to males that were independent of maternal care. The emergence of the sex effect in the basal HPA axis function during adolescence is unsurprising, considering that a defining feature of adolescence is puberty and an underlying spike in both gonadal hormones and CORT secretion [74,75,76]. While the relationship between the HPA and HPG axes is complex, there is evidence that increases in estradiol during puberty could be responsible for HPA axis activation, while testosterone is considered to be HPA-inhibiting [77,78]. Prior work examining sex differences in basal CORT in human adolescents, however, has been ambiguous [75,79,80]; thus, the strong sex effect (*p* = 0.002) reported in this study should help provide some clarity to this literature. This result supports a biological basis for the vulnerability of females to stress-related psychiatric disorders, as well as for the tendency of such disorders to emerge during adolescence [81,82].

On the other hand, the sex by maternal care interaction on diurnal CORT secretion at 12 months is rather curious and difficult to explain. This is partially complicated by the fact that this sex difference both emerges and is abolished prepubertally, precluding an effect of puberty-related increases in gonadal steroid levels, which remain relatively constant and low during the HPG-quiescent juvenile period. A potential explanation for this observation could be related to mini-puberty, which is a brief, primarily perinatal period during which the HPG axis is active at quasi-pubertal levels. In humans, this neuroendocrine surge has largely ceased by the first year, but follicle-stimulating hormone (FSH) remains elevated in females until 2–4 years (developmentally equivalent to 6–12 months in rhesus monkeys) [83]. Thus, the basal CORT levels in control females at this age could be related to residual FSH elevations from mini-puberty. This would explain why the effect disappears by 18 months, when FSH has dropped to normal prepubertal levels. It would also explain why basal CORT levels in MALT females overlap with those of the males at 12 months. GCs are generally HPG axis suppressive. If maltreatment-induced CORT elevations inhibit FSH release, then the neuroendocrine profile of MALT females would likely be largely indistinguishable from that of males at this age. A major caveat of this explanation is that no evidence currently exists to suggest that FSH impacts the HPA axis either directly or indirectly, though considering the extensive intertwining of the HPA and HPG axes, it is not an unreasonable speculation. Studies involving FSH manipulations in gonadally suppressed subjects could be used to test this hypothesis in the future. This is a relatively understudied area of research with potentially major implications, as interruption of mini-puberty by ELA could have serious developmental outcomes.

An alternate explanation could be accelerated biological aging in individuals with ELA experiences, as previously supported by shortened telomere length [84,85], and accelerated maturation of amygdala/limbic functional connectivity [86], and unpublished data from our MALT model]. The diurnal CORT rhythm in MALT females at 12 months resembles that of the control females at 18 months, suggesting that maltreatment might speed up biological maturation of this neuroendocrine axis. In any case, the disruption of both basal activity and negative feedback may serve as a double hit on HPA axis function that predisposes maltreated females toward psychopathology.

While this study followed a rigorous and careful experimental design to address limitations in prior work and human studies, including the random assignment of infants at birth to experimental groups to control for confounding effects of heritable factors, it is not without its limitations. For one, the MALT exhibited by the mothers was not uniform. While minimum threshold criteria existed to qualify a mother as maltreating, above that threshold there was variability in the frequency and severity of physical abuse and rejection received by MALT infants that may account for some of the variability in our results. Additionally, the total plasma CORT measurements reflect both free (active, typically 10% of total CORT) plasma CORT and CORT bound to carrier proteins, such as corticosteroid-binding globulin (CBG, 80%) and albumin. The degree of CORT binding by CBG determines how much CORT is available to exert actions at tissues, and there is evidence that CBG levels are influenced by both sex and stress [87,88,89]. Free CORT can be analyzed in other biological samples, such as saliva and urine, but such samples are subject to methodological constraints that conflicted with the time-sensitive nature of the measurements used in this study. CBG levels could not be immediately determined from the samples due to a lack of availability of reliable quantitative assays that have been validated in this species. An additional limitation was that DEX treatment during adolescence resulted in a ceiling effect of CORT suppression with no escape at PM (midday) in any animal from any group. The DEX dose used in this study (0.25 mg/kg BW) is the same that has previously worked with great sensitivity to detect escape from DEX suppression in younger rhesus monkeys (infants and juveniles; [60,61]), but it was likely too high for adolescent animals; this was probably due to lower liver metabolic rates in older animals that result in slower clearance of the drug from the blood. Finally, this animal model of maternal maltreatment does not, and cannot, replicate the multi-faceted and complex social and environmental human experiences. The findings reported within this paper intentionally highlight the commonalities that we found to be related to the HPA axis in these two species, but it is important to consider these results carefully as the developmental milieu of the rhesus macaque throughout its lifespan is considerably different to that of humans, and therefore other social, psychological, cultural, cognitive, and biological factors may affect the human trajectory compared to rhesus macaques.

## 5. Conclusions

Our findings suggest that infant maltreatment causes protracted, but transient, dysregulation of the HPA axis across the first year of life, as evidenced by alterations in basal CORT and GR-mediated negative feedback. The exclusively female increase in diurnal CORT rhythm in the juvenile period indicates that females are particularly vulnerable to stress-induced HPA axis perturbations. The observed effects of maltreatment disappear by the mid-juvenile period, suggesting that the impact of maltreatment on HPA axis function may be transient. Our findings regarding HPA axis function normalization during the pre-pubertal and adolescent rhesus stages are consistent with human evidence of recalibration/normalization of HPA axis function during adolescence in children that switch from adverse/deprived environments to supportive adoptive families [66]. Altogether, this research has broad implications regarding the developmental course and biological processes that lead from ELA to psychopathology and possible pathways to resiliency.

## Data Availability

Data can be requested from the corresponding authors.
